# Travel Distance to Dialysis and Mortality Among Hemodialysis Patients in a Geographically Small Country

**DOI:** 10.1007/s10900-025-01496-0

**Published:** 2025-07-01

**Authors:** Chen Namimi-Halevi, Lital Keinan-Boker, Rita Dichtiar, Pazit Beckerman, Michal Bromberg

**Affiliations:** 1 Department of Epidemiology and Preventive Medicine, School of Public Health, Gray Faculty of Medical and Health Sciences, Tel Aviv University, Tel Aviv, Israel; 2 Israel Center for Disease Control, Israel Ministry of Health, Sheba Medical Center, Tel Hashomer, 5265601 Ramat Gan, Israel; 3 School of Public Health, Faculty of Social Welfare and Health Sciences, University of Haifa, Haifa, Israel; 4 The Institute of Nephrology and Hypertension, Sheba Medical Center, Tel-Hashomer, Ramat Gan, Israel; 5 School of Medicine, Gray Faculty of Medical and Health Sciences, Tel-Aviv University, Tel Aviv, Israel

**Keywords:** Health disparities, Geographic accessibility, Travel distance, Chronic disease, Hemodialysis, Health equity

## Abstract

Evidence on the association between dialysis facility accessibility and mortality in small countries is limited. This study evaluated the association between travel distance and one- and two-year mortality among Israeli end-stage renal disease (ESRD) patients. Data were obtained from the National Renal Replacement Therapy Registry, encompassing all Israeli ESRD patients aged ≥ 45 years who initiated hemodialysis in 2010–2021. Sociodemographic (age, sex, population group), treatment-related (hospital/community treatment, primary renal disease, incident-year cohort), and geographic (residency socioeconomic and peripherality indices) data were collected. Travel distances from the most recent address to the initial treating facility were categorized into three groups based on the 50th and 90th percentiles, excluding outliers (> 100 km). Adjusted Cox regressions assessed associations between travel distances and mortality, with incident-year cohort-specific models evaluating temporal hazard variations. Analyses included 15,606 patients. The long-distance group (> 26.39 km) was characterized by younger age, Arab ethnicity, peripheral residence, and lower socioeconomic status. Compared to the short-distance group (≤ 6.80 km), the hazard ratios (HRs) in the intermediate-distance group (> 6.80 and ≤ 26.39 km) were 1.216 (1.106–1.337) for one-year and 1.181 (1.093–1.275) for two-year mortality. In the long-distance group, the HRs were 1.718 (1.460–2.021) for one-year and 1.554 (1.351–1.787) for two-year mortality (*P-for-trend between travel distances* <.001). Sensitivity and temporal analyses confirmed consistent associations across incident-year cohorts, alternative cut-offs, and outlier inclusion. The association between travel distance and mortality highlights the need to improve geographic accessibility to healthcare to reduce inequities, especially for life-sustaining chronic treatments such as hemodialysis.

**Supplementary Information:**

The online version contains supplementary material available at 10.1007/s10900-025-01496-0.

## Introduction

Geographic accessibility to healthcare services is a pivotal element in ensuring equitable health for all [1, 2]. Research highlights the associations between geographic proximity to healthcare resources and improved patient engagement, adherence to treatment protocols, and overall health status [3]. This concept gains even greater significance when applied to conditions of a chronic nature, frequent necessity, and life-sustaining importance, such as in-center hemodialysis, which remains the dominant renal replacement therapy for end-stage renal disease (ESRD) patients [4]. As the prevalence of treated ESRD rises [5], mainly due to enhanced survival rates, and the incidence age for dialysis treatment initiation progressively increases [6], the aging of the growing patient population underscores the need for accessible dialysis services.

Prior studies have documented variations in travel distances to dialysis centers within countries [7–9]. Several factors, including socioeconomic status (SES), race and sex, have been associated with decreased access to dialysis services [8, 10]. For individuals undergoing hemodialysis, poor proximity to treatment facilities has been linked to diminished quality of life [11]. This is due to the added physical strain, time consumption, and financial costs associated with prolonged travel [11]. Limited prior research has examined the associations between travel distance and health outcomes among hemodialysis patients. Remote residence has been associated with a higher incidence of ESRD-related complications, such as anemia [12]. Studies by Ajmal et al. (2016) [13] and Thomson et al. (2012) [14] have reported higher mortality rates among remote ESRD patients in the United States, with similar findings observed by Moist et al. (2008) in an international study [11].

To the best of our knowledge, to date, the research exploring the associations between travel distances to dialysis centers and health outcomes has predominantly been conducted in large countries [11, 13, 14], characterized by extensive geographic areas. In these settings, significant travel distances pose notable barriers to accessing quality care, potentially impacting treatment outcomes. However, there remains a gap in understanding these associations within smaller countries, where travel distances are generally shorter. Israel serves as a pertinent example with its compact area of 22,072 km^2^ [15] and 79 dialysis centers [16], suggesting potentially lesser obstacles to treatment accessibility. On the other hand, there are further potential barriers, including traffic congestion and limited availability of public transportation services [17–19], which may add another layer of complexity to ensuring adequate dialysis access, even within shorter travel distances.

Therefore, this study aims to investigate the associations between travel distances to hemodialysis facilities and mortality among adult ESRD patients in Israel, using a comprehensive and ongoing national database and adjusting for potential confounders, while also assessing whether these associations have varied over time.

## Methods

Data were derived from the National Israeli Renal Replacement Therapy Registry [20], managed by the Israel Center for Disease Control under the Israeli Ministry of Health [21], which serves as a comprehensive data repository regarding all patients receiving any form of renal replacement therapy in Israel. This registry relies on mandatory reporting by the dialysis centers and health funds, as well as reports from the National Transplant Center [22], supporting accurate, reliable, and comprehensive data collection.

**Fig. 1:**
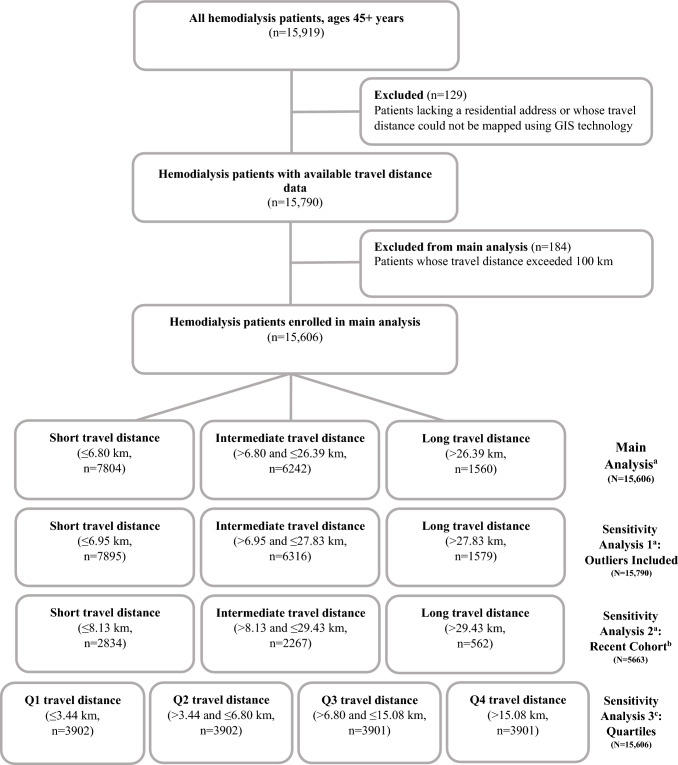
Flowchart of study participants. ^a^The classification is based on the 50th and 90th percentiles; ^b^The most recent cohort refers to the 2018-2021 cohort; ^c^The classification is based on quartiles. GIS, Geographic Information System

This historical cohort study included all Israeli ESRD patients aged 45 and older who initiated hemodialysis as their primary dialysis treatment from 2010 to 2021. Only patients receiving in-center hemodialysis were included, as this modality requires frequent facility visits. Patients for whom no residential address was available, or whose travel distance could not be determined using Geographic Information System (GIS) technology [23], were excluded. Finally, patients traveling more than 100 km to their dialysis facility were also excluded from the main analysis; this cutoff was chosen after confirming that the farthest distance from any patient's address to the nearest facility was 100 km, ensuring that the threshold accurately represented genuinely extreme travel conditions, as this scenario is improbable. A sensitivity analysis was conducted including all patients, as well as outliers (Fig. 1).

### One- and Two-Year Mortality

Dates of dialysis initiation, modality transitions to peritoneal dialysis, facility transfers, and withdrawals from hemodialysis were extracted from the registry data. All-cause mortality dates were sourced from records of the Israeli Ministry of Interior [24], and kidney transplantation dates were provided by the National Transplant Center. Follow-up for participants was commenced at dialysis initiation and concluded at the earliest event of death, kidney transplantation, modality change, facility transfer, withdrawal, or the study's end (after one or two years from dialysis initiation). Outcomes were coded as deceased (1) or censored for non-mortality reasons (0). Patients who transferred to a different treatment institute were censored, as re-enrolling these patients with updated travel distances post-transfer would not allow follow-up from initiation of dialysis treatment.

### Travel Distance

Patients’ addresses were obtained through a one-time cross-referencing of ID numbers from registry data with the Ministry of Interior's records in 2024, using the most recent address available at the time of cross-referencing. The first treating institute and its address were sourced from the registry and the Ministry of Health data, respectively. The quickest routes to the treating dialysis centers were determined via GIS modeling. Patients were categorized into short (≤ 50th percentile), intermediate (> 50th and ≤ 90th percentiles), and long travel distance groups (> 90th percentile), using a percentile-based stratification approach previously described [13]. This approach was intended to capture a dose–response relationship, and the selected cutoffs reflected the rationale that differences at shorter distances were not expected to produce meaningful effects, whereas dividing at the median would yield a more substantial contrast. In addition, using the 90th percentile as an upper cut-off allowed for adequate representation of individuals at the extremes. An additional sensitivity analysis incorporated quartile-based cutoff.

### Additional Variables

#### Socio-Demographic Variables 

Age at the start of dialysis (years), sex (male/female), and population group (Arabs, Jews and others; The “Others” category encompasses individuals who do not identify as Jewish or Arab. This includes non-Arab Christians, members of other religions, and individuals without a religious classification [25]) were obtained from the registry data.

#### Geographic Variables 

These variables were derived either directly from patients’ addresses or indirectly by translating these addresses into small Statistical Geographic Areas (SGA):

*Peripherality:* Patients’ addresses were cross-referenced with Central Bureau of Statistics (CBS) [26] data to calculate a peripherality index [27]. The value of the index is a weighted sum of two components: (A) A potential accessibility index, measuring the proximity of the patient’s local authority to all other local authorities in Israel adjusted for their population sizes; (B) Proximity to the border of the Tel Aviv district, highlights the patient’s local authority’s proximity to the economic and business center of Israel. Patients were categorized based on their residence's peripherality into peripheral (1–3), intermediate (4–6), and central (7–10) groups.

*SES:* SGA data were cross-referenced with Points Location Intelligence (PLI) [28] data to determine the SES within patients'residential areas. The PLI's SES index is based on the CBS socio-economic index, which assesses demographic composition, education, living standards, employment, and pensions, and is regularly updated to improve accuracy [29]. Patients were classified into low (1–3), medium (4–6), and high (7–10) SES groups.

#### Treatment-Related Variables

These were based on registry data and included the following:

*Incident-year cohort:* Patients were categorized into three cohorts, each spanning four years, based on the year they commenced their treatment (2010–2013, 2014–2017, 2018–2021). A sensitivity analysis was conducted including only patients from the most recent cohort (2018–2021), thereby minimizing the potential impact of residential relocation between treatment initiation and the single cross-referencing with Ministry of the Interior data in 2024.

*Facility type:* Patients were classified according to the location of their treatment facility, whether within hospitals or in community settings.

*Primary renal disease:* Dialysis centers report the initial diagnosis of renal disease at the start of treatment using the European Dialysis and Transplant Association (EDTA) codes [30]. Patients were grouped based on their underlying renal condition: (a) diabetes mellitus (types 1 and 2), (b) hypertension and renal vascular disease, (c) glomerulonephritis, (d) other, (e) unknown/missing.

**Statistical analysis:** Statistical analysis was performed using SPSS software, version 29. Continuous variables were presented as means (standard deviations) or medians (quartiles), and categorical variables as frequencies (percentages).

Differences in sociodemographic, treatment-related, and geographic characteristics of study participants by travel distance were evaluated using ANOVA/Kruskal–Wallis tests (for continuous variables) and Chi-square tests (for categorical variables). P-for-trend analyses were conducted to assess linear trends across the travel distance categories, using the Mantel–Haenszel test for categorical variables and Spearman’s rank correlation for continuous variables. One- and two-years survival differences by travel distance were analyzed using the Log-rank test and Kaplan–Meier survival curves. Collinearity among study's variables was assessed using Spearman’s rank correlation test, with collinearity defined as a Spearman correlation coefficient ≥ 0.5. No collinearity was detected among the different potential variables. Additionally, no significant interactions were found between travel distance and the variables SES, peripherality, and population group on the hazard of mortality.

One- and two-year mortality were compared between the study's different travel distance categories by Cox proportional hazards regressions, adjusted for identified confounders (enter mode). P-for-trend analyses across travel distance categories were conducted by incorporating travel distance as an ordinal variable in the regression models. In the multivariable models, missing values for SES (284 out of 15,606), peripherality (59 out of 15,606), and population group (6 out of 15,606) were excluded due to their small, negligible proportions. Conversely, the substantial number of missing/unknown primary renal disease diagnoses (2541 out of 15,606) was retained as a separate category within the regression. No missing values were observed for the remaining covariates.

Additionally, three sensitivity analyses were conducted as mentioned above.

To evaluate the consistency of the association across different time periods, Cox proportional hazards models were also used to calculate hazard ratios (HRs) separately for each four-year incident-year cohort between 2010 and 2021. Interaction was assessed by introducing an interaction term between travel distance (as both a continuous and ordinal variable, in separate models) and incident-year cohort into the full model, and testing the statistical significance of the interaction term.

All statistical tests were two-tailed, with a significance level set at p < 0.05.

**Ethics:** This study was granted an exemption from ethical approval by the Israeli Ministry of Health’s Ethics Committee, as it addressed health policy issues in accordance with the mandate of the Ministry of Health. Informed consent was not required, as the study was based on a data registry. To protect patients'privacy, the dataset used for analyses did not include any identifying details, such as names, identification numbers, or home addresses. Additionally, exact dates of key events, such as treatment initiation or death, were not included; instead, only the number of days from treatment initiation to these events was recorded.

## Results

Among the 15,606 patients who initiated hemodialysis treatment between 2010 and 2021, the median travel distance to initial treating dialysis facility was 6.80 km (Q1 = 3.43, Q3 = 15.08). The patients’ mean age was 70.19 ± 10.96 years, with about two-thirds of the cohort being male and roughly 20% of Arab ethnicity. The majority of patients resided in central areas, and approximately 50% lived in medium SES regions. The distribution across the incident-year cohorts was relatively balanced, with the largest group belonging to the most recent cohort (2018–2021). Approximately 70% of patients began treatment in a hospital setting, and diabetes mellitus was the most frequently reported underlying renal disease (Table 1).

**Table 1:** Characteristics of Study's Participants, by Travel Distance to Initial Dialysis Facility Categories^a^, 2010–2021

Variable	N	All (N = 15,606)	Short Travel Distance (N = 7804)	Intermediate Travel Distance (N = 6242)	Long Travel Distance (N = 1560)	P-Value
***Sociodemographic variabsle***						
*Age, mean years ± standard deviation*	15,606	70.19 ± 10.96	71.26 ± 10.81	69.30 ± 11.03	68.46 ± 10.94	** <.001**
*P-for-Trend*						** <.001**
*Age group, n (%)*	15,606					** <.001**
45–64		5000 (32.0)	2213 (28.4)	2200 (35.3)	587 (37.6)	
65–74		4906 (31.4)	2464 (31.6)	1944 (31.1)	498 (31.9)	
75 +		5700 (36.5)	3127 (40.1)	2098 (33.6)	475 (30.5)	
*P-for-Trend*						** <.001**
*Sex, n (%)*	15,606					0.39
Female		5632 (36.1)	2794 (35.8)	2290 (36.7)	548 (35.1)	
Male		9974 (63.9)	5010 (64.2)	3952 (63.3)	1012 (64.9)	
*P-for-Trend*						0.85
*Population group, n*** (%)**	15,600					** <.001**
Jews and Others^b^		12,439 (79.7)	6821 (87.5)	4462 (71.5)	1156 (74.1)	
Arabs		3161 (20.3)	978 (12.5)	1779 (28.5)	404 (25.9)	
*P-for-Trend*						** <.001**
***Geographic Variables***						
*Travel distance, median Km (Q1, Q3)*	15,606	6.80 (3.43, 15.08)	3.44 (2.31, 4.79)	13.00 (9.41, 17.62)	35.93 (30.50, 46.60)	** <.001**
*P-for-Trend*						** <.001**
*Socioeconomic status, n (%)*	15,322					** <.001**
Low		3633 (23.7)	1703 (22.2)	1592 (25.9)	338 (22.5)	
Medium		7245 (47.3)	3626 (47.3)	2793 (45.4)	826 (55.0)	
High		4444 (29.0)	2340 (30.5)	1767 (28.7)	337 (22.5)	
*P-for-Trend*						** <.001**
*Peripherality, n (%)*	15,547					** <.001**
Peripheral		2740 (17.6)	517 (6.6)	1462 (23.5)	761 (49.7)	
Intermediate		2277 (14.7)	1258 (16.1)	766 (12.3)	253 (16.5)	
Central		10,530 (67.7)	6029 (77.3)	3985 (64.1)	516 (33.7)	
*P-for-Trend*						** <.001**
***Treatment-related variables***						
*Incident-year cohort, n (%)*	15,606					** <.001**
2010–2013		4725 (30.3)	2481 (31.8)	1825 (29.2)	419 (26.9)	
2014–2017		5218 (33.4)	2724 (34.9)	2054 (32.9)	440 (28.2)	
2018- 2021		5663 (36.3)	2599 (33.3)	2363 (37.9)	701 (44.9)	
*P-for-Trend*						** <.001**
*Facility type, n (%)*	15,606					** <.001**
Community		4557 (29.2)	2715 (34.8)	1502 (24.1)	340 (21.8)	
Hospital		11,049 (70.8)	5089 (65.2)	4740 (75.9)	1220 (78.2)	
*P-for-Trend*						** <.001**
*Primary renal disease, n (%)*	15,606					**0.004**
Glomerulonephritis		691 (4.4)	352 (4.5)	270 (4.3)	69 (4.4)	
Diabetes Mellitus		8200 (52.5)	4016 (51.5)	3394 (54.4)	790 (50.6)	
Hypertension/Renal Vascular Disease		2063 (13.2)	1080 (13.8)	779 (12.5)	204 (13.1)	
Other		2111 (13.5)	1027 (13.2)	847 (13.6)	237 (15.2)	
Unknown/Missing		2541 (16.3)	1329 (17.0)	952 (15.3)	260 (16.7)	
*P-for-Trend*						0.40

Categorical variables are expressed as n (%); Continuous variables are expressed as mean ± standard deviation or median (Q1, Q3 quartiles)

Percentages represent the proportion of patients in each travel distance category who belong to the given subgroup

P-values with bold font indicates statistical significance (p < 0.05)

^a^Travel distance categories were classified based on the 50th (6.80 km) and 90th (26.39 km) percentiles

^b^The “Others” category encompasses individuals who do not identify as Jewish or Arab. This includes non-Arab Christians, members of other religions, and individuals without a religious classification

### Patient’s Characteristics by Travel Distance Category

In the main analysis, cut-offs were set at 6.80 km (50th percentile) and 26.39 km (90th percentile) with median travel distances of 3.44, 13.00, and 35.93 km for the short, intermediate, and long travel distance groups, respectively. Patients traveling longer distances were characterized by younger age and a higher likelihood of having low-to-medium SES and residing in peripheral areas. The proportion of Arab patients was higher in the intermediate- and long-distance groups compared to the short-distance group. The distribution of sex was similar across the travel distance groups. Additionally, patients from the most recent cohort accounted for a larger proportion of the long-distance group. Although a statistically significant difference was observed in the distribution of primary renal diseases across travel distance groups, no clinically meaningful differences were identified, with diabetes mellitus remaining the dominant underlying disease in all groups (Table 1).

### Unadjusted Survival Probabilities

**Fig. 2:**
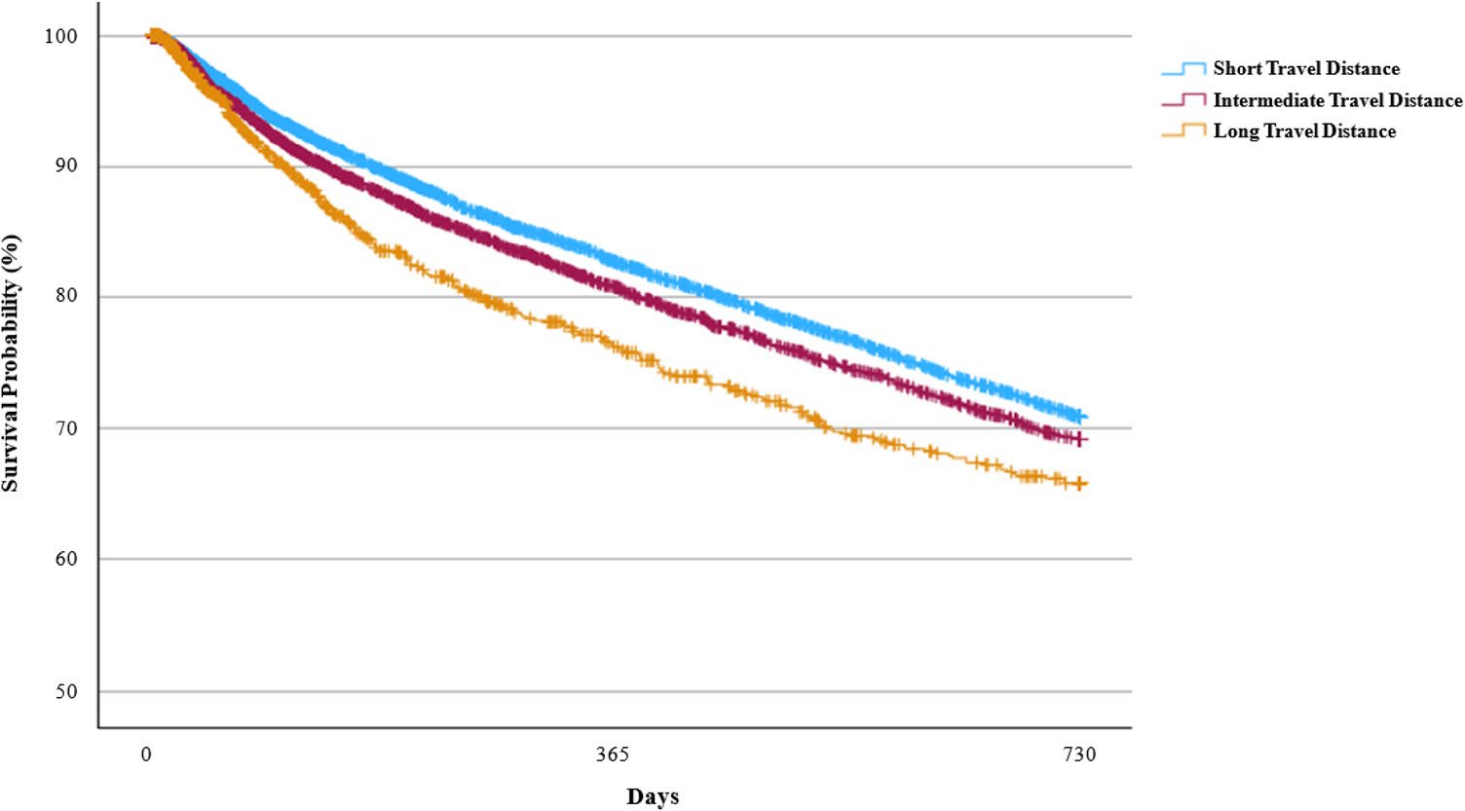
Kaplan–Meier Survival Curves for One-^a^ and Two^b^-Year Survival by Categories of Travel Distance to Initial Dialysis Facility.^c^, 2010–2021 (N = 15,606). ^a^One-Year Survival Probability (%, 95% Confidence Interval): Short travel distance: 82.71% (81.76%, 83.62%). Intermediate travel distance: 80.77% (79.54%, 81.94%). Long travel distance: 76.22% (73.16%, 79.00%). Log-rank test: p<.001. ^b^Two-Year Survival Probability (%, 95% Confidence Interval): Short travel distance: 70.89% (69.72%, 72.03%). Intermediate travel distance: 69.21% (67.72%, 70.65%). Long travel distance: 65.84% (62.26%, 69.17%). Log-rank test: p<.001. ^c^Travel distance was categorized according to the 50th (6.80 km) and 90th (26.39 km) percentiles into: short (n=7804), intermediate (n=6242), and long (n=1560) travel distances.

A statistically significant difference (p < 0.001) in survival probabilities was observed across the three travel distance categories for both one-year and two-year survival (Fig. 2). Survival probabilities were highest in the short-distance group (82.71% at one year, 70.89% at two years), followed by the intermediate-distance group (80.77% at one year, 69.21% at two years), and lowest in the long-distance group (76.22% at one year, 65.84% at two years).

Sensitivity Analyses 1 (including outliers) and 2 (limited to the 2018–2021 cohort) demonstrated statistically significant results as well. In Sensitivity Analysis 3 (quartiles-based classification), survival probabilities were similar in the first and second quartiles, lower in the third quartile, and lowest in the fourth quartile (p < 0.05 across all sensitivity analyses) (Supplementary Figs. 1a-c).

### Multivariable Models

In the main analysis, after adjusting for sociodemographic, clinical, and geographic variables, statistically significant differences in mortality were observed across the travel distance categories for both one-year and two-year outcomes (p < 0.001) (Table 2). Patients in the short-distance group served as the reference category. For one-year mortality, the HRs (95% CI) were 1.216 (1.106–1.337; p < 0.001) for the intermediate-distance group and 1.718 (1.460–2.021; p < 0.001) for the long-distance group. For two-year mortality, the HRs were 1.181 (1.093–1.275; p < 0.001) for the intermediate-distance group and 1.554 (1.351–1.787; p < 0.001) for the long-distance group. Linear trends across travel distance categories were statistically significant in both models (p < 0.001).

**Table 2:** Adjusted^a^ Cox Proportional Hazards Regression Results for the Associations Between Travel Distance to Initial Dialysis Facility and One- and Two-Year Mortality (Main and Sensitivity Analyses)

		One-year mortality		Two-year mortality	
Outcome: mortality	N*	adjusted^a^ hazard ratio (95% confidence interval)	P-value	adjusted^a^ hazard ratio (95% confidence interval)	P-value
***Main analysis***^b^	15,306		** <.001**		** <.001**
Short travel distance (≤ 6.80 km)		Reference		Reference	
Intermediate travel distance (> 6.80 and ≤ 26.39 km)		1.216 (1.106, 1.337)	** <.001**	1.181 (1.093, 1.275)	** <.001**
Long travel distance (> 26.39 km)		1.718 (1.460, 2.021)	** <.001**	1.554 (1.351, 1.787)	** <.001**
*P-for-Trend*			** <.001**		** <.001**
***Sensitivity analysis 1***^b^ ***(Outliers included)***	15,484		** <.001**		** <.001**
Short travel distance (≤ 6.95 km)		Reference		Reference	
Intermediate travel distance (> 6.95 and ≤ 27.83 km)		1.236 (1.124, 1.359)	** <.001**	1.194 (1.106, 1.289)	** <.001**
Long travel distance (> 27.83 km)		1.623 (1.376, 1.915)	** <.001**	1.441 (1.249, 1.662)	** <.001**
*P-for-Trend*			** <.001**		** <.001**
***Sensitivity analysis 2***^b^ ***(2018–2021 Cohort)***	5558		**0.013**		**0.002**
Short Travel Distance (≤ 8.13 km)		Reference		Reference	
Intermediate travel distance (> 8.13 and ≤ 29.43 km)		1.254 (1.046, 1.503)	**0.015**	1.217 (1.050, 1.411)	**0.009**
Long travel distance (> 29.43 km)		1.470 (1.053, 2.053)	**0.024**	1.530 (1.158, 2.022)	**0.003**
*P-for-Trend*			**0.003**		** <.001**
***Sensitivity analysis 3***^c^ ***(Quartiles-based)***	15,306		** <.001**		** <.001**
Q1 travel distance (≤ 3.44 km)		Reference		Reference	
Q2 travel distance (> 3.44 and ≤ 6.80 km)		0.978 (0.867, 1.103)	0.72	0.964 (0.877, 1.060)	0.45
Q3 travel distance (> 6.80 and ≤ 15.08 km)		1.173 (1.041, 1.322)	**0.009**	1.132 (1.028, 1.246)	**0.012**
Q4 travel distance (> 15.08 km)		1.433 (1.254, 1.636)	** <.001**	1.341 (1.202, 1.496)	** <.001**

^a^The main analysis, as well as Sensitivity Analyses 1 and 3, were adjusted for age group, sex, population group, socioeconomic status, peripherality, incident-year cohort, facility type, and primary renal disease. Sensitivity Analysis 2 was adjusted for these same factors, with the exception of the incident-year cohort

^b^Travel distance categories were classified based on the 50th and 90th percentiles

^c^Travel distance categories were classified based on quartiles

P-values with bold font indicates statistical significance (p < 0.05)

^*^N represents the number of observations included in the model. The following n observations were excluded due to missing data in one or more covariates: Main Analysis = 300; Sensitivity Analysis 1 = 306; Sensitivity Analysis 2 = 105; Sensitivity Analysis 3 = 300

The sensitivity analyses demonstrated similar results, showing a dose–response relationship between travel distance and one- and two-year mortality. In Sensitivity Analysis 3, no mortality differences were observed between the first and second travel distance quartiles (Table 2).

### Temporal Variation Analysis

Similar multivariate analyses for each four-year cohort separately also demonstrated a dose–response relationship between the three travel distance categories of the main analysis and one- and two-year mortality (Supplementary Table 1). For one-year mortality, adjusted HRs ranged from 1.210 to 1.260 for intermediate versus short travel distances, and from 1.470 to 1.912 for long versus short travel distances. For two-year mortality, adjusted HRs ranged from 1.201 to 1.224 for intermediate versus short travel distances, and from 1.398 to 1.681 for long versus short travel distances.

Interaction analyses between incident-year cohort and travel distance for both one-year and two-year mortality revealed no statistically significant interactions, whether travel distance was examined as a continuous variable (p-for-interaction = 0.53 and 0.79 for one- and two-year mortality, respectively) or as an ordinal variable (p-for-interaction = 0.37 and 0.64, respectively).

## Discussion

The spatial distribution of healthcare facilities plays a crucial role in determining communities’ ability to access essential care [31], particularly for chronic conditions requiring frequent treatments, such as hemodialysis [4]. This study identified a significant association between longer travel distances to treating dialysis centers and increased one- and two-year mortality risk among ESRD patients, with a significant dose–response relationship. These findings remained consistent over time. Notably, this is the first study to examine this association using national data in a country with a relatively small geographic area and shorter travel distances.

The sensitivity analyses conducted in this study reinforced the primary analysis’ conclusions. Nevertheless, in the quartile-based analysis, no significant difference in mortality was observed between patients in the first and second quartiles of travel distance. This may indicate that the association between travel distance and mortality becomes evident only beyond a certain threshold of travel distances, plausibly due to higher impact on overall travel time and effort invested in the commute [32]. Moreover, this sensitivity analysis also supports the travel distance categorization used in the primary analysis, based on the median and the 90th percentile.

Temporal variation analyses further confirmed that the association between travel distance and mortality remained consistent over time. The persistent association may suggest that changes in patient survival and in travel-related barriers, such as road congestion, have evolved in parallel and proportionally across all travel distance groups—resulting in a stable association that neither weakened nor intensified. Notably, in Israel, road congestion has increased considerably; Between 2010 and 2021, total vehicular travel increased by 28% and the number of registered vehicles rose by 49%, whereas road infrastructure expanded by only 11% in total length and 22% in total surface area [33]. Similar dynamics have been observed in other countries as well. During the same period, one-year survival among dialysis patients in Israel increased from 80.2% to 82.8%, and two-year survival from 67.2% to 70.5%, according to data from the Israeli National Renal Replacement Therapy Registry (data not shown) [20].

The incidence rate of ESRD treatment initiations in Israel is among the highest globally [16, 34], with 91.6% of prevalent patients receiving in-center hemodialysis [35], underscoring the substantial burden of this treatment within the country. Since 1995, Israel has offered dialysis under its National Health Insurance (NHI) free of charge, ensuring every resident with ESRD can receive the necessary treatment without cost [34]. According to the NHI law, medical services shall be given ‘…with reasonable quality, within a reasonable timeframe and at a reasonable distance from the patient’s residence, subject to the financial resources available to the Sick Fund’ [36]. However, the definition of ‘a reasonable distance’ remains vague.

Nowadays, GIS is increasingly utilized in decision-making processes for locating medical facilities, establishing itself as a vital strategic tool in public health planning. Traditionally, GIS has been employed primarily to ensure reasonable access to healthcare services [37]. However, findings from this study suggest a need to move beyond the goal of reasonable to optimal accessibility [38], as even relatively small disparities in travel distance may impact survival and mortality outcomes, in addition to the evident effects on quality of life.

It is important to note that hemodialysis patients traveling longer distances were also characterized by lower SES, peripheral residence and Arab minority ethnicity. These findings emphasize the disparities in accessibility of vulnerable populations, further underscoring the issue of health inequities within Israel's relatively small geographic size [39, 40].

Previous studies have investigated the associations between travel distance and health outcomes across a range of healthcare services. A systematic review by Kelly et al. synthesized findings from 108 studies examining various healthcare fields, including dialysis, oncology, women’s health services, chronic disease management, psychiatric care, elective surgeries, and general healthcare [3]. The “distance decay” effect—where greater travel distances were consistently associated with poorer health outcomes, such as lower survival rates, delayed care, and reduced follow-up adherence—was observed in 77% of the studies.

In-center hemodialysis is unique compared to other healthcare services, as it requires very frequent travel to the treatment facility, several times per week, and it is life-sustaining [4]. Several previous studies have examined the association specifically in dialysis patients. A study by Moist et al., conducted as part of the Dialysis Outcomes and Practice Patterns Study (DOPPS) in various countries (including the United States, Canada, Australia, and the United Kingdom), investigated the association between self-reported travel time to dialysis center and patient mortality over a follow-up period of 2–4 years. The study found a 20% higher risk of death in the > 60-min group compared to the ≤ 15-min group, with a significant trend across all travel categories [11].

Thompson et al. examined the association among 726,347 hemodialysis patients in the United States over a 13-year period (median follow-up: 2.7 years). Distance was measured as the shortest driving distance from the patient’s residence to the nearest dialysis center. Their findings revealed that patients living more than 100 miles (~ 160 km) from a dialysis center faced a 21% higher risk of death compared to those residing within 10 miles (~ 16 km). The trend of increasing mortality risk with greater driving distance was statistically significant [14].

Ajmal et al. studied the association among 181,349 ESRD patients in the United States (2007–2008). Distances were calculated using straight-line methods based on patient and nearest dialysis facility ZIP codes and were categorized into quartiles. Results indicated that patients living in urban areas at a distance of 18 miles (~ 29 km) or more had an 8% higher two-year mortality than their urban counterparts living 0–3.3 miles (up to ~ 5.3 km) from a dialysis facility [13].

There are several differences between the current study and those reviewed, including the countries studied (their healthcare systems and their geographic sizes), travel distance categories investigated, the facility used for distance measurement (treating vs. nearest facility), methods of distance calculation (shortest travel distance vs. straight-line or self-reported travel times, exact addresses vs. ZIP codes), confounders adjustments, handling of censoring events, the years studied, and the length of follow-up. Despite these differences, all studies consistently show that reduced geographic accessibility is linked to higher mortality among hemodialysis patients.

The association between travel distance and mortality among dialysis patients has several potential explanations. Longer travel distances are associated with an increased likelihood of missed or shortened dialysis sessions, which compromise the effectiveness of life-sustaining treatment [8, 9, 11, 12]. Transportation challenges, particularly for those reliant on external services, exacerbate this issue [3, 14]. It is also possible that patients living farther away are offered less intensive dialysis treatment plans, either due to logistical constraints or lower expectations of adherence [11]. Additionally, greater distances can result in delays in accessing emergency care [12], potentially worsening outcomes during critical health events. The physical and mental burden of frequent, lengthy travel may contribute to chronic fatigue and diminished quality of life, further impacting overall health [11]. Patients living farther from dialysis centers may also face barriers to receiving timely or optimal care, including limited access to high-quality facilities or specialized services [8, 12]. Furthermore, distance from a dialysis center can serve as a proxy for reduced access to other essential healthcare services, either due to geographical constrains or lack of time due to the frequent long commute to the dialysis center [9, 12], compounding the challenges faced by these patients, which are elderly and have high probability of comorbidities, such as diabetes [41, 42].

This study faces several limitations, mitigated through various measures. First, there is a potential selection bias from excluded outliers that was addressed through sensitivity analyses that included these outliers. Patients without residential addresses represented a negligible proportion, reducing concerns about such bias. Second, information bias may arise from discrepancies between the updated addresses used in this study and patients’ actual residences at dialysis initiation. To mitigate this, the study included only patients aged ≥ 45—who are less likely to relocate—excluded travel distance outliers in the main analysis, and conducted sensitivity analyses focusing on the most recent cohort, alongside a temporal variation analysis to ensure consistency of the results. Third, SES and peripherality data were cross-referenced once in 2024 and may not capture changes over time; however, the general stability of these variables likely minimizes their impact on the findings. Fourth, SES is an ecological variable based on place of residence, but its use in this context is widely accepted. Fifth, although the models were adjusted for multiple confounders, residual confounding remains possible due to limited clinical information in the registry. Lastly, while travel distance may differ from travel time, it is a more stable measure, as it is not affected by temporal fluctuations in traffic conditions—such as differences between hours of the day or between weekdays and weekends. Though travel times likely increased during the study period, as the growth in vehicular travel exceeded the expansion of the road network, it is assumed that this occurred proportionally across all travel distance groups, as reflected by the lack of change in HRs over time in the study.

Nevertheless, this study also has several strengths. It relies on a comprehensive, ongoing national registry, spanning 12 years and including over 15,500 patients. The registry provides detailed information on changes in treatment status, such as facility transfers, shifts in treatment modality, treatment cessation, or kidney transplantation, enabling precise tracking of individual patients and accurate assessment of their contribution time to the study. The registry also offers complete and accurate mortality data, ensuring reliable outcome measurement. Furthermore, it includes data on the actual treating facility and its accurate address, a key parameter which was absent in similar studies. Additionally, distance was calculated using the road network, which has been shown to correlate more closely with actual travel time compared to straight-line distance, as used in other studies. Temporal variation analysis was performed, alongside several sensitivity analyses, which incorporated changes in the study population and variations in selected cut-offs.

In conclusion, this study found that patients living farther from their treating dialysis center face an increased risk of mortality compared to those residing nearby, even in a country where travel distances are relatively moderate. Furthermore, it implies the potential existence of a threshold, below which this association does not hold. This study highlights a significant health inequity, alongside the inherent diminishment in quality of life associated with such circumstances, which may act as a barrier also to a broader range of essential medical services. Policymakers should take action to mitigate this association and address accessibility inequities. This can be achieved through strategic planning for the establishment of dialysis centers with a focus on minimizing disparities or by promoting and supporting home-based treatment modalities, alongside enhancement of the accessibility of other healthcare services essential for dialysis patients. Additionally, promotion of health literacy among these patients is required, emphasizing the importance of timely medical evaluation, follow-up, and treatment even beyond the dialysis context. Further research is needed to validate and expand upon these findings. Additionally, it is recommended to adopt this method for examining associations in other healthcare services, particularly those requiring frequent travel and of significant therapeutic importance.

## Supplementary Information

Below is the link to the electronic supplementary material.

Supplementary file1 (DOCX 289 KB)

Supplementary file2 (DOCX 16 KB)

## Data Availability

The data supporting this study are not publicly available due to privacy and confidentiality restrictions.

## Abbreviations

CBS: Central Bureau of Statistics
ESRD: End-Stage Renal Disease
EDTA: European Dialysis and Transplant Association
GIS: Geographic Information System
HR: Hazard Ratio
NHI: National Health Insurance
PLI: Points Location Intelligence
SES: Socioeconomic Status
SGA: Statistical Geographic Areas
