# Supplementary material for: Travel Distance to Dialysis and Mortality Among Hemodialysis Patients in a Geographically Small Country
Source: J Community Health. 2025 Jul 1;50(6):1115–26. doi: 10.1007/s10900-025-01496-0 (PMC12586237; doi:10.1007/s10900-025-01496-0)
Supplement: Supplementary file 1 — Supplementary file1 (DOCX 289 KB) [file 10900_2025_1496_MOESM1_ESM.docx]

**Supplementary Figure** **1a.** Kaplan-Meier Survival Curves for One-^a^ and Two^b^-Year Survival by Categories of Travel Distance to Initial Dialysis Facility^c^, 2010-2021**:** Sensitivity Analysis 1 (Outliers Included)

**
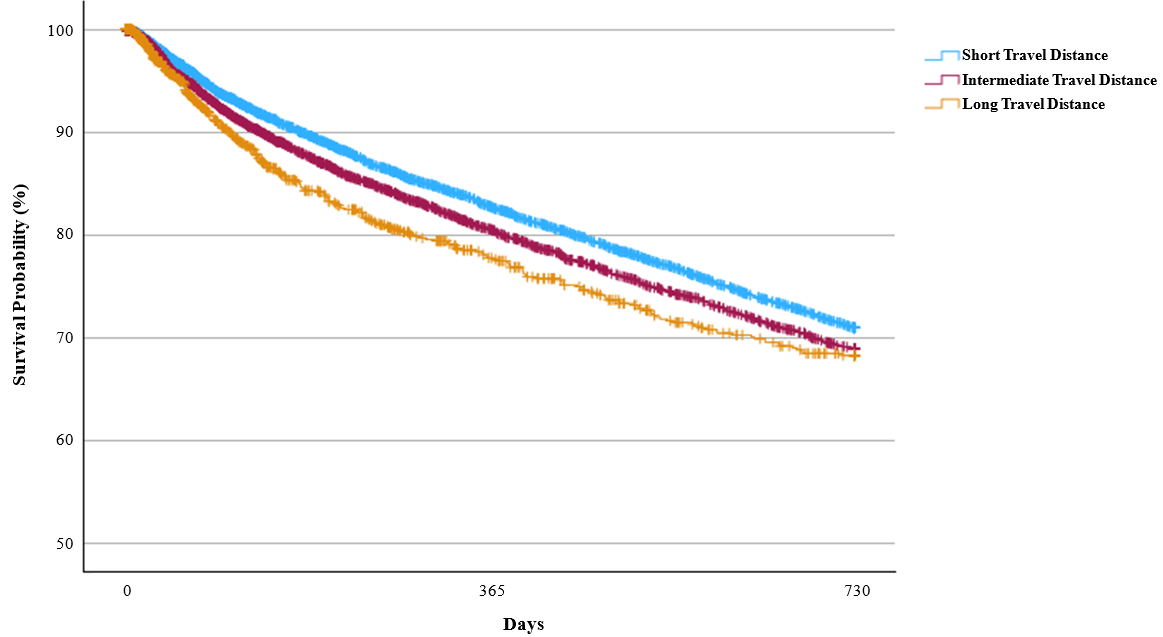
**

**^a^One-Year Survival Probability (%, 95% Confidence Interval):**

- Short travel distance: 82.80% (81.85%, 83.70%)
- Intermediate travel distance: 80.54% (79.30%, 81.71%)
- Long travel distance: 77.76% (74.77%, 80.44%)
- Log-rank test: p<.001

**^b^Two-Year Survival Probability (%, 95% Confidence Interval):**

- Short travel distance: 70.95% (69.79%, 72.08%)
- Intermediate travel distance: 68.95% (67.45%, 70.39%)
- Long travel distance: 68.26% (64.73%, 71.53%)
- Log-rank test: p=0.002

^c^Travel distance categories were classified based on the 50th (6.95 km) and 90th (27.83 km) percentiles.

**Supplementary Figure** **1b.** Kaplan-Meier Survival Curves for One-^a^ and Two^b^-Year Survival by Categories of Travel Distance to Initial Dialysis Facility**^c^:** Sensitivity Analysis 2 (Cohort 2018-2021)


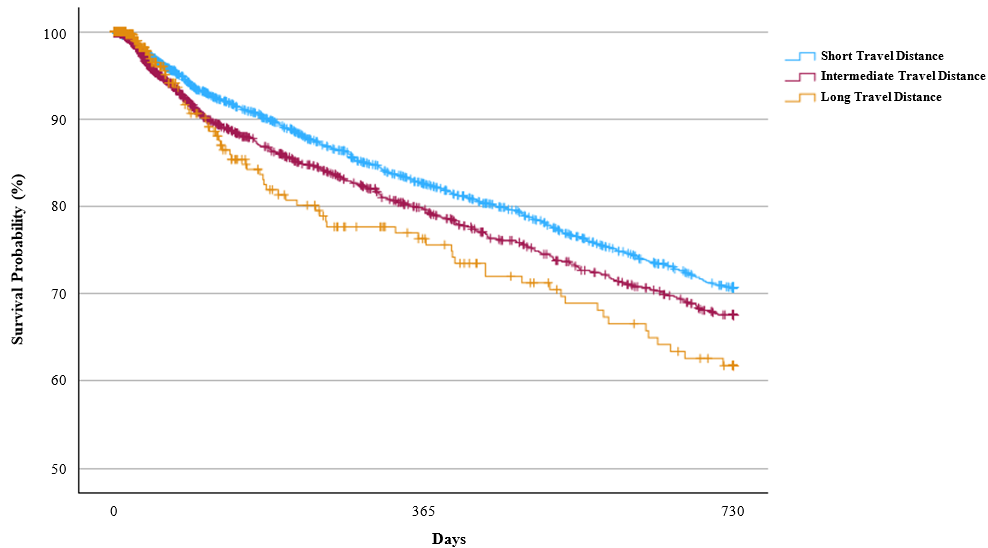


**^a^One-Year Survival Probability (%, 95% Confidence Interval):**

- Short travel distance: 82.62% (80.86%, 84.24%)
- Intermediate travel distance: 79.64% (77.04%, 81.99%)
- Long travel distance: 76.30% (69.50%, 81.79%)
- Log-rank test: p=0.016

**^b^Two-Year Survival Probability (%, 95% Confidence Interval):**

- Short travel distance: 70.72% (68.56%, 72.76%)
- Intermediate travel distance: 67.57% (64.43%, 70.51%)
- Long travel distance: 61.76% (53.56%, 68.94%)
- Log-rank test: p=0.010

^c^Travel distance categories were classified based on the 50th (8.13 km) and 90th (29.43 km) percentiles.

**Supplementary Figure** **1c.** Kaplan-Meier Survival Curves for One-^a^ and Two^b^-Year Survival by Categories of Travel Distance to Initial Dialysis Facility^c^, 2010-2021**:** Sensitivity Analysis 3 (Quartiles-Based Classification)

**
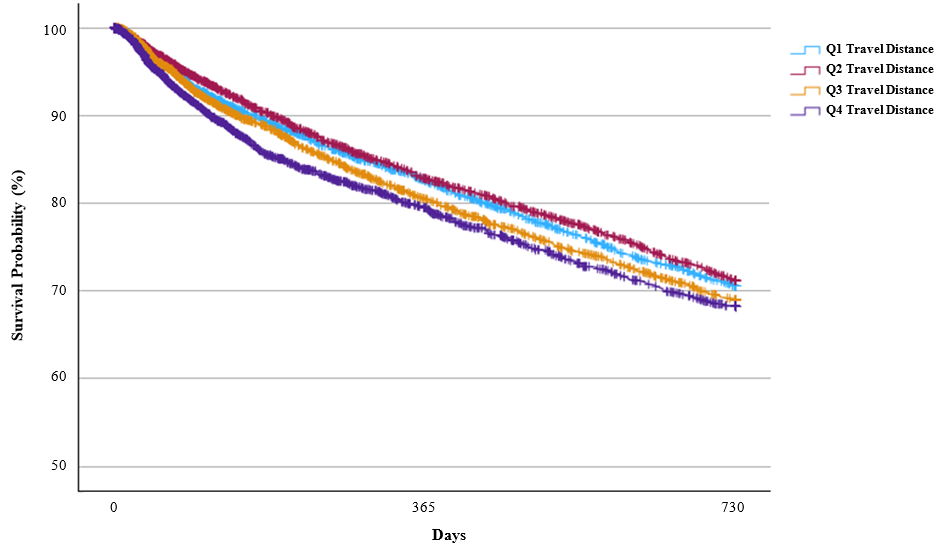
**

**^a^One-Year Survival Probability (%, 95% Confidence Interval):**

- Q1 travel distance: 82.61% (81.27%, 83.86%)
- Q2 travel distance: 82.82% (81.44%, 84.11%)
- Q3 travel distance: 80.50% (78.98%, 81.92%)
- Q4 travel distance: 79.49% (77.74%, 81.12%)
- Log-rank test: p<.001

**^b^Two-Year Survival Probability (%, 95% Confidence Interval):**

- Q1 travel distance: 70.60% (68.96%, 72.17%)
- Q2 travel distance: 71.20% (69.50%, 72.82%)
- Q3 travel distance: 69.01% (67.19%, 70.75%)
- Q4 travel distance: 68.26% (66.13%, 71.29%)
- Log-rank test: p=0.005

^c^Travel distance categories were classified based on quartiles (3.44 km, 6.80 km, 15.08 km).
