# Supplementary material for: Travel Distance to Dialysis and Mortality Among Hemodialysis Patients in a Geographically Small Country
Source: J Community Health. 2025 Jul 1;50(6):1115–26. doi: 10.1007/s10900-025-01496-0 (PMC12586237; doi:10.1007/s10900-025-01496-0)
Supplement: Supplementary file 2 — Supplementary file2 (DOCX 16 KB) [file 10900_2025_1496_MOESM2_ESM.docx]

**Supplementary Table 1.** Adjusted^a^ Cox Proportional Hazards Regression Results for the Associations Between Travel Distance to Initial Dialysis Facility^b^ and One- and Two-Year Mortality, Stratified by Incident-Year Cohort

|  |  | **One-Year Mortality** |  | **Two-Year Mortality** |  |
| --- | --- | --- | --- | --- | --- |
| **Outcome: Mortality** | **N*** | **Adjusted**^a^ **Hazard Ratio**  **(95% Confidence Interval)** | **P-value** | **Adjusted**^a^ **Hazard Ratio**  **(95% Confidence Interval)** | **P-Value** |
| **Years: 2010-2013** | 4627 |  | **<.001** |  | **<.001** |
| Short Travel Distance  (≤6.29 km) |  | Reference |  | Reference |  |
| Intermediate Travel Distance  (>6.29 and ≤25.00 km) |  | 1.210 (1.029, 1.423) | **0.021** | 1.224 (1.074, 1.396) | **0.003** |
| Long travel Distance  (>25.00 km) |  | 1.912 (1.469, 2.488) | **<.001** | 1.681 (1.333, 2.119) | **<.001** |
| *P-for-Trend* |  |  | **<.001** |  | **<.001** |
| **Years: 2014-2017** | 5121 |  | **<.001** |  | **0.002** |
| Short Travel Distance  (≤6.36 km) |  | Reference |  | Reference |  |
| Intermediate Travel Distance  (>6.36 and ≤24.59 km) |  | 1.260 (1.080, 1.471) | **0.003** | 1.201 (1.060, 1.360) | **0.004** |
| Long Travel Distance  (>24.59 km) |  | 1.696 (1.298, 2.217) | **<.001** | 1.398 (1.112, 1.759) | **0.004** |
| *P-for-Trend* |  |  | **<.001** |  | **<.001** |
| **Years: 2018-2021** | 5558 |  | **0.013** |  | **0.002** |
| Short Travel Distance  (≤8.13 km) |  | Reference |  | Reference |  |
| Intermediate Travel Distance  (>8.13 and ≤29.43 km) |  | 1.254 (1.046, 1.503) | **0.015** | 1.217 (1.050, 1.411) | **0.009** |
| Long Travel Distance  (>29.43 km) |  | 1.470 (1.053, 2.053) | **0.024** | 1.530 (1.158, 2.022) | **0.003** |
| *P-for-Trend* |  |  | **0.003** |  | **<.001** |

^a^All models were adjusted for age group, sex, population group, socioeconomic status, peripherality, facility type, and primary renal disease.

^b^Travel distance categories were classified based on the 50th and 90th percentiles.

P-values with bold font indicates statistical significance (p<0.05).

*N represents the number of observations included in the model. The following n observations were excluded due to missing data in one or more covariates: Years 2010-2013 = 98; Years 2014-2017 = 97; Years 2018-2021 = 105.
